# Author’s reply to Rao et al.—“DEGRO practical guidelines: radiotherapy of breast cancer-regional nodal irradiation”

**DOI:** 10.1007/s00066-026-02610-4

**Published:** 2026-10-08

**Authors:** Kai Borm, Marciana Nona Duma, David Krug, Juliane Hörner-Rieber

**Affiliations:** 1 Department of Radiation Oncology, TUM School of Medicine and Health, Technical University of Munich (TUM), Klinikum rechts der Isar, Munich, Germany; 2 Department of Radiation Oncology, Helios Clinics of Schwerin, University Campus of MSH Medical School Hamburg, Schwerin, Germany; 3 Department for Human Medicine, MSH Medical School Hamburg, Hamburg, Germany; 4 Department of Radiotherapy and Radiation Oncology, University Hospital Schleswig-Holstein, Christian Albrechts University Kiel, Kiel, Germany; 5 Department of Radiation Oncology, University Hospital Düsseldorf, Düsseldorf, Germany

To the Editor,

We thank Rao et al. for their comments on our updated DEGRO practical guidelines on regional nodal irradiation for breast cancer [1] and for their Letter to the Editor. We highly appreciate their perspective, particularly regarding the challenges of implementing evidence-based guidelines in low- and middle-income countries.

We would like to address the three main concerns raised in their letter:

## Trial heterogeneity and risk stratification for RNI

The discussion on the optimal treatment of nodal low-risk disease is indeed controversial. We agree that the existing data do not unequivocally answer the question of how to treat patients with low-risk N+ disease. A recent survey among international ESTRO experts demonstrated the broad range of data interpretation and clinical practice [2]. In our current recommendation, we present a consensus of the German expert panel, acknowledging that the existing data allow for alternative interpretations.

Considering the overall survival (OS) benefit of internal mammary node (IMN) irradiation in the DBCG IMN 2 trial [3]—where modern treatment techniques and systemic therapies were used and no subgroups were identified for safe omission of IMN irradiation (IMNI)—and the recently published 20-year follow-up of the EORTC 22922 trial [4], which showed a benefit in terms of breast cancer-specific mortality even in pN0 patients, the proposal by Rao et al. to restrict comprehensive RNI to patients with > 3–4 positive lymph nodes appears, however, difficult to justify.

Regarding risk stratification and the option of limiting axillary irradiation to the caudal regions of levels I and II, we defined this low-risk cohort in the text based on the following clinicopathological factors: one positive lymph node, ≤ T1, Extracapsular Extension (ECE)-negative, HR-positive, lateral tumor location, age > 50 years. Given the lack of high-level evidence to guide patient selection, these criteria are intended as pragmatic clinical guidance rather than strong evidence-based recommendations.

## Methodological consistency in trial selection

We wish to clarify that we did not disregard the 10-year results of the SUPREMO trial [5] due to methodological inconsistencies. Rather, we cautioned against using its results to argue against the need for RNI in patients with intermediate-risk breast cancer after mastectomy. Although approximately 75% of patients had node-positive disease, only 10% received supraclavicular irradiation and just 1% underwent IMN irradiation. Consequently, the trial primarily evaluated postmastectomy chest wall irradiation rather than contemporary comprehensive RNI.

The NSABP B‑51/RTOG 1304 [6] trial, on the other hand, explicitly focused on the impact of RNI. We fully acknowledge the limitations of this study and addressed them in our manuscript, including the markedly lower-than-expected event rate and the limited follow-up time. Nevertheless, this randomized study provides the strongest evidence to date in this setting; thus, in our view, it should not be disregarded. The findings from NSABP B‑51/RTOG 1304 are further supported by the 10-year data from the RAPCHEM registry [7]. Both trials excluded patients with locally advanced breast cancer, which is why we stated that radiotherapy de-escalation based on treatment response should not be extrapolated to patients with cT4 and/or cN2‑3 breast cancer.

We agree with Rao et al. that recommendations surrounding axillary management after neoadjuvant chemotherapy are continuously evolving, which is why we highlighted the fact that further evidence is required and that results from ongoing trials, such as TAXIS and ALLIANCE A11202, are eagerly awaited.

## Resource-stratified implementation in lMICs and DIBH

Our guidelines were developed to reflect the current standard of care and available technologies within Germany and comparable healthcare systems. We acknowledge that extrapolating these recommendations to settings where, e.g., complete axillary lymph node dissection remains the practical baseline and deep-inspiration breath-hold (DIBH) is not always feasible due to resource constraints, requires pragmatic adaptation.

However, we would like to emphasize the importance of DIBH, which in most cases leads to a significant reduction in organ-at-risk doses (even if the IMN is not irradiated). The 20-year data from the EORTC 22922 trial [4] showed improvements in breast cancer-related mortality; however, this was offset by an increase in other-cause mortality, which only appeared after 15 years. It is reasonable to assume that this non-breast-cancer-related mortality can be reduced by implementing modern radiotherapy techniques, which have led to a dramatic reduction in OAR doses compared to the techniques used in the EORTC 22922 trial [8, 9]. Deep-inspiration breath-hold is a crucial tool in this endeavor to improve the benefit-to-risk ratio of regional nodal irradiation. This is especially important since radiotherapy is a highly cost-effective treatment. Performed as voluntary DIBH without the need for specialized equipment, DIBH has been shown to yield comparable OAR doses and setup reproducibility [10].

We kindly ask the authors to appreciate that adapting the recommendations of a German expert panel to LMICs, while undoubtedly important, is beyond the scope of the present consensus statement. We would, however, strongly encourage Rao et al., given their expertise and their in-depth understanding of the available infrastructure and resources in LMICs, to develop a dedicated consensus tailored to these settings, which would undoubtedly provide valuable guidance for clinicians practicing in these environments.

## Data Availability

All data supporting the findings of this work are available from the corresponding author upon reasonable request.

## References

[1] Borm K et al (2026) DEGRO practical guidelines: radiotherapy of breast cancer-regional nodal irradiation. Strahlenther Onkol 202(7):692–707 10.1007/s00066-026-02554-9 PMC13290887 42289007

[2] Putora PM et al (2025) What guides regional nodal irradiation in node positive breast cancer following upfront sentinel lymph node biopsy? Radiother Oncol 211:111055 10.1016/j.radonc.2025.111055 40695422

[3] Nielsen AWM et al (2025) Internal mammary node irradiation in 4541 node-positive breast cancer patients treated with newer systemic therapies and 3D-based radiotherapy (DBCG IMN 2): a prospective, nationwide, population-based cohort study. Lancet Reg Health Eur 49:101160 10.1016/j.lanepe.2024.101160 PMC11732476 39810969

[4] Kaidar-Person O et al (2026) Internal mammary chain and medial supraclavicular lymph node irradiation in stage I-III breast cancer (EORTC trial 22922/10925): an unplanned subset analysis of 20-year outcomes in patients with node-negative breast cancer. Lancet Oncol 27(7):817–829 10.1016/S1470-2045(26)00233-0 42372743

[5] Kunkler IH et al (2025) Ten-Year Survival after Postmastectomy Chest-Wall Irradiation in Breast Cancer. N Engl J Med 393(18):1771–1783 10.1056/NEJMoa2412225 PMC7618363 41191939

[6] Mamounas EP et al (2025) Omitting Regional Nodal Irradiation after Response to Neoadjuvant Chemotherapy. N Engl J Med 392(21):2113–2124 10.1056/NEJMoa2414859 PMC12269788 40466065

[7] de Wild SR et al (2022) De-escalation of radiotherapy after primary chemotherapy in cT1-2N1 breast cancer (RAPCHEM; BOOG 2010-03): 5‑year follow-up results of a Dutch, prospective, registry study. Lancet Oncol 23(9):1201–1210 10.1016/S1470-2045(22)00482-X 35952707

[8] Siman-Tov M et al (2024) Dosimetric Analyses of the Three Radiation Techniques Used in the EORTC 22922/10925 IM-MS Breast Cancer Trial. Clin Oncol (R Coll Radiol) 36(11):690–700 10.1016/j.clon.2024.08.012 39261237

[9] Holt F et al (2024) Estimated Doses to the Heart, Lungs and Oesophagus and Risks From Typical UK Radiotherapy for Early Breast Cancer During 2015-2023. Clin Oncol (R Coll Radiol) 36(9):e322–e332 10.1016/j.clon.2024.05.002 PMC11511668 38853062

[10] Bartlett FR et al (2013) The UK HeartSpare Study: randomised evaluation of voluntary deep-inspiratory breath-hold in women undergoing breast radiotherapy. Radiother Oncol 108(2):242–247 10.1016/j.radonc.2013.04.021 23726115

